# Head Protection Device for Individuals at Risk for Head Injury due to Ground-Level Falls: Single Trauma Center User Experience Investigation

**DOI:** 10.2196/54854

**Published:** 2024-03-19

**Authors:** Susan Haag, John Kepros

**Affiliations:** 1 Scottsdale Osborn Medical Center Scottsdale, AZ United States

**Keywords:** health care interventions and technologies, user experience research, usability, brain injury, ground-level fall (GLF), head protection device (HPD), fall risk, patient compliance

## Abstract

**Background:**

Falls represent a large percentage of hospitalized patients with trauma as they may result in head injuries. Brain injury from ground-level falls (GLFs) in patients is common and has substantial mortality. As fall prevention initiatives have been inconclusive, we changed our strategy to injury prevention. We identified a head protection device (HPD) with impact-resistant technology, which meets head impact criteria sustained in a GLF. HPDs such as helmets are ubiquitous in preventing head injuries in sports and industrial activities; yet, they have not been studied for daily activities.

**Objective:**

We investigated the usability of a novel HPD on patients with head injury in acute care and home contexts to predict future compliance.

**Methods:**

A total of 26 individuals who sustained head injuries, wore an HPD in the hospital, while ambulatory and were evaluated at baseline and 2 months post discharge. Clinical and demographic data were collected; a usability survey captured HPD domains. This user experience design revealed patient perceptions, satisfaction, and compliance. Nonparametric tests were used for intragroup comparisons (Wilcoxon signed rank test). Differences between categorical variables including sex, race, and age (age group 1: 55-77 years; age group 2: 78+ years) and compliance were tested using the chi-square test.

**Results:**

Of the 26 patients enrolled, 12 (46%) were female, 18 (69%) were on anticoagulants, and 25 (96%) were admitted with a head injury due to a GLF. The median age was 77 (IQR 55-92) years. After 2 months, 22 (85%) wore the device with 0 falls and no GLF hospital readmissions. Usability assessment with 26 patients revealed positive scores for the HPD post discharge regarding satisfaction (mean 4.8, SD 0.89), usability (mean 4.23, SD 0.86), effectiveness (mean 4.69, SD 0.54), and relevance (mean 4.12, SD 1.10). Nonparametric tests showed positive results with no significant differences between 2 observations. One issue emerged in the domain of aesthetics; post discharge, 8 (30%) patients had a concern about device weight. Analysis showed differences in patient compliance regarding age (χ_1_^2^=4.27; *P*=.04) but not sex (χ_1_^2^=1.58; *P*=.23) or race (χ_1_^2^=0.75; *P*=.60). Age group 1 was more likely to wear the device for normal daily activities. Patients most often wore the device ambulating, and protection was identified as the primary benefit.

**Conclusions:**

The HPD intervention is likely to have reasonably high compliance in a population at risk for GLFs as it was considered usable, protective, and relevant. The feasibility and wearability of the device in patients who are at risk for GLFs will inform future directions, which includes a multicenter study to evaluate device compliance and effectiveness. Our work will guide other institutions in pursuing technologies and interventions that are effective in mitigating injury in the event of a fall in this high-risk population.

## Introduction

Frailty in aging is represented by a decline in functioning, with a risk of poor outcomes, including falls, which have implications for clinical practice and public health [1]. Falls are the primary cause of injury-related death in aging adults as 33% of adults 65 years and older fall each year [2,3].

Falls also represent a large percentage of hospitalized older patients as they may result in multiple injuries, including head trauma [4-6]. A head injury can be a common cause of disability and mortality and may be as mild as a bump, bruise (contusion), or cut and can be moderate to severe due to a concussion. Head injury may lead to premature nursing home admissions and increased hospital length of stay (LOS) with undesirable results for patients and hospitals [7,8]. Due to the aging population worldwide, the incidence of falls will continue to rise [9,10].

Studies have shown a clear pattern of increased health care costs associated with falls and frail individuals and various fall prevention initiatives have been promoted. Of the fall prevention interventions studied, some results have been favorable, such as those with well-developed educational programs [11]. However, others have been inconclusive [12-14], prompting our center to include head injury prevention, and therefore, we investigated a head protection device (HPD), similar to a helmet. In many fields, such as construction and sports, helmets have shown efficacy in preventing head injury risks, especially moderate to severe head injury [15-17]. The human head is vulnerable to even moderate impact as it can cause injury or death. A greater emphasis has been placed on job safety in industries like construction particularly to protect the head from injury, and hard hats and helmets have been required [18]. However, historically, helmets have not been used for normal daily living.

Health care systems are increasingly looking for contexts that provide accessible and efficient care and for medical devices and interventions to improve the patient experience and health outcomes [19,20]. Human factors, a scientific discipline, is important in clinical practice as it reveals how humans interact with interventions, such as devices, regarding expectations and limitations. User experience (UX) focuses on having a deep understanding of users and what they need and value [21-23]. UX research has been used to ascertain user domains such as adherence, usability, and perceived impact and has assisted with intervention development and refinement [24]. Adopting a UX research design will help ensure that new devices are easy to use and meet the needs of most patients.

Clinical practices should target effective strategies that improve individuals’ quality of life and independence including screenings and interventions to manage injuries associated with falls [25,26]. Screenings that measure activities of daily living (ADLs) are essential, as the ability to perform daily tasks safely without exhaustion is a critical component of healthy aging, thus allowing older individuals to maintain their independence and quality of life [27]. Measurement of daily activities is important as these may be predictors of early admission to assisted care facilities or the need for alternative living arrangements [28,29].

Recent literature advocates change toward tailored interventions that preserve an individual’s independence by promoting furthering advancements in evidence-based treatment options and identifying cost-effective strategies [2,3]. Due to an increasing incidence of head injuries after ground-level falls (GLFs) in our trauma center, we designed a study that examined the effects of a low-cost HPD that has the potential to prevent head injury due to a fall.

The purpose of this UX research was to assess compliance by investigating the usability of an HPD from a patient’s perspective in both acute care (hospital) and home contexts. We hypothesized that consented patients would follow the research protocol as recommended and wear the device in the hospital and at the 2 months post discharge. The primary limitation in an aging population is compliance, which we approached first. This in-hospital and home-based UX investigation concerning a low-cost treatment option may serve clinicians to better manage frailty and mitigate injury due to falls in their clinical practice.

## Methods

### Study Design

We considered the UX of frail individuals at this developmental, exploratory stage of a device to examine patient adherence and use. The UX assessment instrument adopted UX domains with a 5-point scale showing a more positive rating (rating of 5) and a lower rating (rating of 1). UX domains included device credibility, satisfaction, usability, adherence, effectiveness, relevance, and aesthetics. The primary outcome variable is patient compliance regarding wearing the device for 2 months. Additional data collected included the frequency of wearing the device during normal daily activities. Consistent with the literature, ADLs (such as ambulating and preparing meals) are critical for independence in aging populations [29].

### Recruitment

Participants were recruited from among patients who were treated at our level 1 trauma center and subsequently admitted to the hospital for observation due to head injury. Protocol inclusion criteria included the following: patients admitted to the hospital with a fall sustaining a head injury, patients with fall risk (eg, patients who fell within the prior year or other physical conditions aligned with fall risk), and patients who were ambulatory and 55 years or older. Head injuries included in the study were patients with a concussion, contusion, lacerations, or loss of consciousness. The individuals recruited did not experience trauma that required surgical intervention. After signing the consent in the hospital, individuals were given an HPD at no cost to wear while ambulatory. After consenting and wearing the HPD for in-hospital observation (and just before discharge), the hospital team asked whether the patients would wear the HPD at home. If the patient agreed, we indicated that the research team would follow up post discharge for additional observations using the UX survey.

### Ethical Considerations

In total, 26 patients, who experienced a fall and sustained a head injury, wore an HPD in hospital, while ambulatory and were evaluated at baseline (before discharge) and at 2 months post discharge. The study protocol was approved by the institutional review board for research ethics and subsequently approved (IRB 1804935). Informed consent was obtained from the 26 patients who met the inclusion criteria and were willing to participate. Confidentiality of information was maintained. The data are anonymized and patients are deidentified. Each patient was assigned a discrete number in the study and data are secured by the research scientist. There was no compensation for patient participation in the study.

### HPD

**Figure 1:**
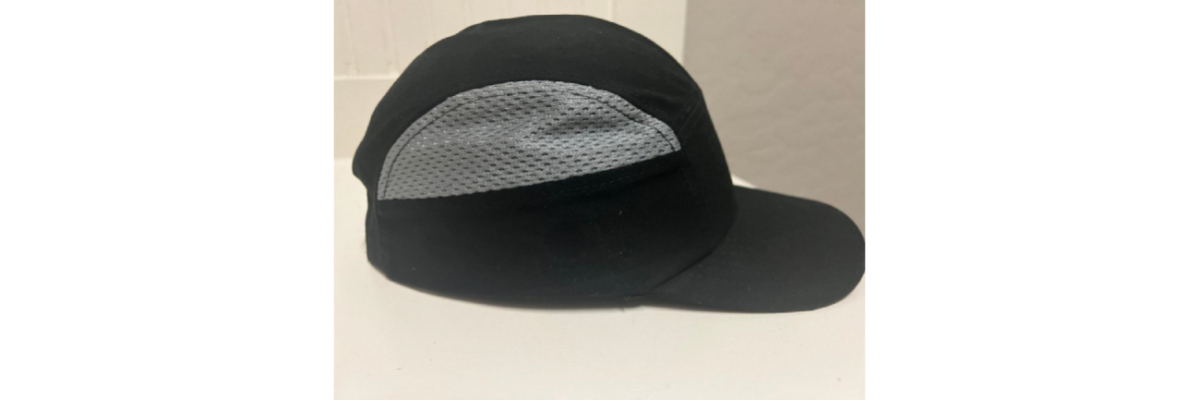
Head protection device.

The HPD includes an impact-resistant technological insert for additional head protection. It helps protect against bumps, scrapes, bruises, and other head injuries. The HPD is designed with ventilation to provide airflow for breathability without compromised protection. The HPD size can be adjusted with a hook and loop strap to give a quick, secure fit. Figure 1 displays the HPD, which looks like a typical baseball cap.

### Usability Survey

A multidisciplinary health care team comprised of physicians, a research scientist, and physical therapists collaborated on the study design, developing a usability survey for patients who are at risk of fall, which led to a tangible and targeted intervention strategy. UX (usability) domain definitions were identified in the literature. Existing domain definitions were examined such as credibility, usability, and satisfaction [24], and additional domains were defined such as effectiveness, relevance, and aesthetics. The domains were refined, used on the usability survey instrument, and functioned as outcome measures. Textbox 1 shows the domains and UX definitions. UX domain data were collected on the instrument using a 5-point scale (5=strongly agree, 4=agree, 3=neutral, 2=disagree, and 1=strongly disagree). Patients were asked if they would recommend the HPD. The survey was intended to evaluate the HPD’s usability and was administered after patients concluded their interaction with the HDP in the hospital. Those who agreed to wear the HPD at home were provided a device and were reevaluated post discharge.

**Domain and user experience definitions.**

- Credibility: whether the user perceives the device to be trustworthy (eg, accuracy and quality of information presented in the patient consent)
- Satisfaction: the user’s overall experience and interaction with the device
- Usability: the user’s perceived ease of use of the device based on technical factors
- Adherence: whether the patient followed the device research protocol and continued to use the device as recommended (compliance) completing outcome measures
- Effectiveness: the extent the user perceives the overall value of the device, including safety and whether they would recommend it to another fall risk individual
- Relevance: the extent to which the device is appropriate for their situation and whether they perceive it meets their needs (provides protection to their head and helps them maintain a sense of independence)
- Aesthetics: factors such as color, pattern, size, shape, and weight

### Data Collection

Quantitative data included demographics (age, sex, and race) and clinical data such as hospital LOS, number of GLFs, readmission to the hospital due to a GLF, and Glasgow Coma Scale. Data were also captured on the usability survey including domains such as device satisfaction, effectiveness, relevance, and aesthetics. Qualitative data were also collected on the usability survey, and patient comments were recorded regarding HPD benefits and opportunities for improvement.

### Statistical Analysis

This UX research methodology included multiple patient observations and differences between observations were examined. Nonparametric tests, used to analyze ordinal and categorical data, were used for intragroup comparisons (Wilcoxon signed rank test). We used descriptive statistics, such that patterns might emerge from the data. Frequencies and percentages are reported for categorical variables. Medians and means with SDs are reported for continuous variables as appropriate. All computations included 26 patients. Group comparisons were made using chi-square tests or Fisher exact tests, where numbers were small and were reported as numbers (%). All variables were assessed for normality. Analyses of categorical variables (age) and patient adherence were tested using the chi-square statistic. Statistical tests are 2-tailed, with a significance level of an α of .05. All statistical analyses were performed using SPSS Statistics for Windows (version 28.0; IBM Corp).

Open-ended patient comments (qualitative data) were analyzed using a 3-step process: data reduction, data display, and conclusion drawing and verification. Data reduction helped sort and compile data excerpts (to organize the data) and assist in developing assertions regarding patient perceptions surrounding wearability (eg, comfort and weight) and modifications of HPD, if necessary. Excerpts were annotated with topics such as the benefits of HPD: positive feedback (aspects recorded as positive by the patient participants regarding HPD experience and interaction) and negative feedback (points considered negative by the patients pertaining to interaction with the device). As a next step, we analyzed the themes that emerged and categorized them based on whether they were related to the usability of the HPD or the health support the device offered.

## Results

### Study Population

Among the 26 participants, 12 (46%) were female and 5 (19%) were non-White, with a median age of 77 (IQR 55-92) years. The average hospital LOS was 3.8 (SD 3.65) days. The majority (n=25, 96%) of patients who experienced head trauma were admitted to the hospital with a head injury due to a GLF (n=1, 4% were other types of falls); 22 (85%) had prior falls in the last 12 months and 16 (62%) had a hospital visit due to a head injury related to a fall within the year; 18 (69%) were on anticoagulants. The mean Glasgow Coma Score was 14.2 (SD 0.44). The age category was divided into 2 groups for analysis: age group 1 comprised of those who were 55 to 77 years and age group 2 comprised of patients 78 years and older.

### Usability Survey Domain Results

In the hospital, all 26 consented patients wore the device with 0 falls recorded. After 2 months, 22 (85%) were wearing the HPD, had 0 falls, and had no hospital readmissions due to GLFs. At 6 months, 16 (62%) patients were compliant with wearing the device, with 0 falls and no hospital readmissions due to a GLF. The results showed positive scores, with no significant differences between ratings in hospital and post discharge regarding device credibility (0.42), satisfaction (0.60), usability (0.80), adherence (0.06), effectiveness (0.53), and relevance (0.09). A difference emerged for the domain of aesthetics. After the discharge, 8 (30%) patients had concerns regarding the device’s weight, saying it was slightly heavier than a typical cap. Overall, users had a positive experience with the HPD and scores revealed that patients felt it was effective and relevant. Thus, post discharge, users would recommend the HPD to others at risk for falls (mean 4.52, SD 0.51). Users were compliant by wearing the device in hospital and at 2 months post discharge, supporting the research hypothesis. Table 1 displays the UX domain means (SDs) for 2 observations.

Differences between categorical variables (age group 1: 55-77 years, group 2: 78 years and older, sex, and race) and protocol adherence were analyzed. Chi-square analysis showed differences in compliance regarding age (χ_1_^2^=4.27; *P*=.04) but not sex (χ_1_^2^=1.58; *P*=.23) or race (χ_1_^2^=0.75; *P*=.60). Age group 1 was more likely to wear the device for normal daily activities.

**Table 1:** In-hospital and postdischarge intragroup domain differences.

User experience domains	Hospital, mean (SD)	Postdischarge, mean (SD)	*P* value
Credibility	3.91 (0.80)	4.01 (0.84)	.42
Satisfaction	4.15 (0.88)	4.80 (0.89)	.60
Usability	4.27 (0.66)	4.23 (0.86)	.80
Adherence	4.50 (0.86)	4.30 (1.06)	.06
Effectiveness (value)	4.62 (0.49)	4.69 (0.54)	.53
Relevance	4.42 (0.75)	4.12 (1.10)	.09
Aesthetics	3.38 (1.30)	2.96 (1.83)	.003

### Patient Device Use in Daily Activities

The usability survey data captured patient device use during typical ADLs at 2 weeks and at 2 months post discharge. Users were provided a list of daily activities and were asked to rate the frequency of wearing the device. Consistent with the literature, ADLs, such as ambulating and preparing meals, are critical for independence in an aging population [29]. The highest score on the usability instrument was a “5” which indicated that the patient would wear the HPD “most often.” In-home contexts, patients indicated they most often wore the device ambulating and when driving (to meals and doctor appointments) and less often for personal hygiene. Table 2 shows within-group differences in device use in daily activities.

**Table 2:** Within-group differences in device use in daily activities.

Daily activities	Two weeks, mean (SD)	Two months, mean (SD)	*P* value
Ambulating	4.31 (0.92)	4.15 (1.12)	.47
Driving (or being driven)	4.04 (0.77)	4.12 (0.76)	.16
Grocery shopping or shopping	3.69 (1.28)	3.58 (1.23)	.54
Relaxing (TV)	4.00 (1.06)	3.31(1.10)	.20
Housekeeping	3.35 (1.09)	3.27 (1.00)	.67
Preparing meals	2.77 (0.99)	2.50 (1.06)	.07
Personal hygiene	2.42 (0.94)	2.27 (0.96)	.49

### Positive Patient Feedback

Open-ended questions on the usability instrument elicited patient qualitative comments regarding HPD benefits and opportunities for improvement. As a result, 2 dominant themes emerged, namely HPD usability and HPD as health support (protection). Usability was associated with the use of the device and functionality in terms of wearability. Health support included themes that were aligned with head protection for a patient.

Usability and relevance from the patients’ perspective translated into wearability, and the majority of patients wore the device after 2 months post discharge. Participants felt that the HPD was comfortable and easy to wear. However, 8 (30%) patients mentioned that the HPD was not as light as a typical cap due to the protective “technology insert” and suggested the HPD could be lighter in weight. One male participant stated,

The cap is heavier than a usual baseball cap and it took me longer to get used to it. I would like it a bit lighter in weight if possible and more air vents to let in air.

Health support from the participant’s perspective sufficed as the primary benefit, as 18 (69%) commented that the device protected their head in the event of a fall. Patients called the device a “cap” as it resembles a baseball cap. One patient stated, “Protection for my head is important. I will wear it going out to eat and to doctor appointments.” Another female participant indicated, “I wear it eight hours a day to protect my head.” Two patients (male and female) indicated post discharge, they hit their heads on cabinets, as 1 commented:

I already bent over and hit my head on a cabinet; it protected me from another head injury. Since wearing the cap, I have not had a fall, only a bump and I had on my cap.

A 74-year-old female participant stated, “I fell last year and I will wear this walking whenever possible. It protects my head.” A male participant noted, “The device is protective and comfortable; I forgot I had it on.” From patient comments, the HPD is cognate with head protection.

## Discussion

### Principal Findings

Using a UX design, we investigated the usability of a novel HPD on patients with head injury in acute care and home contexts to predict future compliance. All 26 patients provided positive scores for the HPD post discharge regarding satisfaction, usability, effectiveness, and relevance. Nonparametric tests showed positive results, with no significant differences between 2 observations at 2 months. Chi-square analysis showed a significant difference in HPD compliance regarding age but not sex or race as age group 1 was more likely to wear the device for normal daily activities. Patients most often wore the device ambulating and head protection was identified as the primary benefit. Thus, patients were most likely to recommend the HPD to others at risk of GLFs.

Due to the consistently high rate of head injuries after GLFs in our center, the targeted team strategy for an HPD and UX research design was developed. We realized that patient compliance in the geriatric population has been a limiting factor and approached that aspect first. Patients adhered to the research protocol by wearing the device in the hospital and post discharge, in the home, supporting the research hypothesis. At 2 months, 22 (85%) patients wore the device with 0 falls recorded and no readmissions due to falls.

Our multidisciplinary team, a diverse group of medical professionals, consisting of physicians, research scientists, and physical therapists, studied a device to be worn during daily activities in home environments. Recent literature has advocated for home care strategies [30] and interventions to be used in home contexts where falls most often occur [31]. Managing falls in this high-risk population is complex, requiring a systemic and collaborative approach directed by a multidisciplinary team focused on improving patient outcomes [3].

### Limitations

Accuracy is critical regarding the collection of patient data, and the in-hospital data collection was conducted under medical supervision. However, the limitations of the UX research included the nature of self-reporting by participants post discharge at 2 and 6 months. One measure to counter this bias was to include a family member during the evaluation to corroborate the patient’s self-reported data and responses. Another issue and limitation, we noted, was the difficulty of trying to reconnect or contact this population at follow-up due to cognitive decline, the extent and severity of head trauma, and other injuries associated with a GLF.

### Conclusions

The results show our proposed HPD intervention will have a high compliance rate in those at risk for GLFs as it was considered usable, protective, and relevant. Managing individuals with fall risk may include future investigations of specific interventions and low-cost devices that preserve a patient’s independence and physical function, and research that contributes to further advancements in evidence-based treatment options. The feasibility and wearability of the device in patients with GLF with head injuries will inform future directions, which includes a multicenter study to evaluate compliance and device effectiveness. Our work will guide other health care institutions in pursuing cost-effective treatments and technological interventions that are usable and effective in improving outcomes for this fall risk population.

## Abbreviations

ADL: activities of daily living
GLF: ground-level fall
HPD: head protection device
LOS: length of stay
UX: user experience
